# Utilization of RESOLV with polymer to produce prazosin hydrochloride nanoparticles and optimization of the process parameters

**DOI:** 10.1038/s41598-024-69128-6

**Published:** 2024-08-07

**Authors:** Eslam Ansari, Bizhan Honarvar, Seyed Ali Sajadian, Zahra Arab Aboosadi, Mehdi Azizi

**Affiliations:** 1 Chemical Engineering Department, Marvdasht Branch, Islamic Azad University, Marvdasht, Iran; 2 Chemical Engineering Department, Faculty of Engineering, University of Kashan, Kashan, 87317-53153 Iran

**Keywords:** Prazosin hydrochloride, RESOLV, Optimization, Taguchi, Polyvinyl pyrrolidone, Chemical engineering, Biogeochemistry, Chemistry, Engineering

## Abstract

In this study, rapid expansion of a supercritical solution into a Liquid Solvent (RESOLV) was used for the first time to produce pharmaceutical nanoparticles of Prazosin hydrochloride (PRH). The Taguchi method (robust design) was utilized to design the experiments and ensure obtaining the optimal process conditions. The pressure (15–25 MPa), temperature (308–328 K) and nozzle diameter (300–700 μm) effects on the morphology and size distribution of the resulting particles were also examined. The size of the particles decreased from about 40 μm to the range of (252–418 nm). FTIR, DLS, FESEM, XRD, DSC were used to characterize the primary and processed PRH particles. According to DSC investigations, RESOLV-produced PRH showed lower crystallinity than original PRH.

## Introduction

In pharmaceutical industries, researchers seek to reduce the particle size of drugs for many reasons. Most pharmaceutical products have weak solubility in aqueous environments due to the solid state of the product particles^1^. The poor solubility of solid drugs in water can lead to problems in oral medications' pharmaceutical formulation and bioavailability, such as the drug absorption percentage in comparison with the initial dose. To improve the bioavailability of drugs and increase the solubility of poorly soluble drugs in water, various methods have been proposed, such as size reduction, liposome, salt formation, prod rugs, complexation with cyclodextrins, emulsions, self-emulsifying formulations, surfactants and pH adjustments^2^. PRH, known as piperazine monohydrochloride 1-(4-amino-6, 7-dimethoxy-2-quinazolinyl)-4-(2-furanylcarbonyl) and formulated as C_19_H_22_ClN_5_O_4_, is a pharmacological selective a1-adrenoceptor antagonist. PRH is commonly known for heart failure treatment and high blood pressure control^3–5^. PRH has very low bioavailability mainly due to its insoluble property in water. As a result, the development of effective methods to increase its solubility and dissolution rate in water has received much attention^6^.

Recently, it has been employed to decrease the drug particles' size, increase their solubility in biological environments, and combine active pharmaceutical ingredients (API) with excipients uniformly^7^. For example, easier absorption and greater activity are facilitated for particles with a smaller diameter, resulting in faster dissolution. Other distinct advantages include longer blood circulating capacity, cell or tissue specific targeting of drugs, less side effects, and higher stability against enzymatic degradation^8–11^. Disadvantages of nano-sized pharmaceutical particles include the difficulty of their production, and the storage and administration of drugs due to the physical instability of nano-sized particles for accumulation^12^. Some mechanical methods, such as crushing, spray drying, freeze drying, milling and grinding, and solute particle recrystallization from solutions utilizing liquid anti-solvents, are employed to reduce particle size^7,13–15^. The destruction of the particle structure due to thermal or mechanical stress is one of the disadvantages of the old methods in the production of nanoscale particles. Other problems of these processes include the production of coarse particles with improper distribution and the presence of other toxic substances or organic solvents in the products^16^. Many studies have examined the application of supercritical carbon dioxide (SC-CO_2_) for drug nanofabrication (micronization) to overcome all these problems and disadvantages^17^.

The supercritical fluid (SCF) technologies are among the solutions proposed to reduce the particle size of pharmaceutical compounds, improving the solubility of weak drugs in water by reducing the size of particles in nano and micro scales and contributing significantly to biochemical and pharmaceutical industries^18^. SC-CO_2_ is particularly popular due to its low viscosity, critical temperature close to ambient, high permeability, inertness, moderate critical pressure, non-flammability, low cost, and non-toxicity^19–21^.

Supercritical carbon dioxide has shown a great ability in various fields including extraction of essential oil^22–24^ , seed oil^24–26^ solubility^27–32^, nanoparticle formation^33–37^, impregnation^38,39^. In addition, SC-CO_2_ can be a solvent proposed for the particle size improvement in drugs^40^. Rapid expansion of supercritical solutions (RESS) among all types of SC-CO_2_ technology is the most efficient and simple way to make nanoparticles, as it produces a product with no or low residual organic solvent and is easy to use^41^. In general, RESS methods containing solutes produce a sudden decrease in SC-CO_2_ dissolution capacity through a small heated orifice^42^. Small particles with a narrow size distribution are formed during rapid expansion in the RESS process, which has been investigated for the production of monocomposite particles^43^. The results obtained from the mechanical investigations of various model solutes carried out using this method confirm the presence of both micron and nano particles in the expansion jet, while micro particles are also obtained as primal products due to effective coagulation and condensation^44^. However, one of the limitations of RESS is the particle size distribution control during free jet expansion, encouraging current efforts to overcome this significant limitation and improve the RESS method. As an example of these developments, SC-CO_2_ solution is rapidly expanded into a liquid solvent (RESOLV), converted into a liquid medium instead of ambient air by SC-CO_2_^45–47^. Coagulation and condensation can be prevented during the particle expansion utilizing a liquid as a receiver throughout the rapid expansion phase, thus producing smaller drug particles by RESOLV compared to RESS^18^. The absence of nano-scale molding agents is one of the unique advantages of the RESOLV method, as the "supercritical fluid rapid expansion process" provides the molding effect, providing deeper insights into the preparation of nanoscale particles^48^.

For example, several techniques were proposed by Reverchon and Adami^49^ using supercritical fluids to produce nanoparticles. Of the unique characteristics found in supercritical fluids lead to more simplicity and flexibility of these processes while imposing less environmental effects. As a result, the obtained nanoparticles are with better potential performance. They also proposed to critically examine supercritical fluid-based methods utilized to produce nanoparticles, nanowires, nanofilms, nanotubes, nanostructured materials, and nanofibers. Pathak et al.^12^ used the RESOLV method to measure ibuprofen nanoparticles in aqueous suspension. Water-soluble polymers with various molecular weights formed the stabilizing agent of the produced nanoparticle suspension. The addition of water-soluble polymers to the aqueous receiving medium in RESOLV aimed to investigate their impact on the developed ibuprofen nanoparticles' profile. Thus, the drug particle morphology and size can be changed at the nanoscale through the choice of stabilizing agent in RESOLV. In a 2008 study, Byrappa et al.^50^ presented a systematic preparation of nanoparticles using supercritical fluid technology with reference to biomedical material processing. Supercritical synthesis of nanomaterials, including carbon nanotubes, magnetic materials, phosphorus, etc., was discussed due to the potential applications of SCF in cases such as cancer treatment, targeted drug delivery systems, bio imaging, neutron absorption therapy, and hyperthermia. Also, detailed discussions have been raised concerning the newer approach to single nanocrystal formation, in-situ surface modification, nanoparticle morphology control, and dispersion. Prasad Rao and Geckeler^51^ reviewed the information provided regarding the techniques available to produce polymeric nanoparticles. It was observed that the advanced technology for the production of attractive polymer nanoparticles required the selection of a suitable method among the various available techniques. According to the obtained results, special methods, such as RESS, RESOLV, and surfactant-free emulsion polymerization, which are environmentally friendly, can be successfully used to increase the properties of nanoparticles in addition to the production of surfactant-free polymer nanoparticles. When micronization is performed using the RESS process, the drug is dissolved with a concentration of about 10^−4^ mol of supercritical carbon dioxide^52^. In a study, Razmimanesh et al.^18^ synthesized sunitinib malate nanoparticles of a supercritical carbon dioxide by an ultrasonically assisted rapid expansion solution into a liquid solvent (US-RESOLV) process. As shown, the best HPMC stabilizer was introduced to increase the drug dissolution rate, and it acted as the best polymer to control the size distribution of polyethylene glycol particles. Comparisons confirmed significantly smaller size of the produced particles (< 600 nm) than untreated sunitinib malate particles. Table 1 presents the findings and some research done in different fields with RESOLV technology. Which shows that the RESOLV technology for the production of PRH nanoparticles is reported for the first time. The study utilized Taguchi-based test design to optimize the PRH particle size reduction. Characterization of the processed and unprocessed particles was conducted utilizing field emission scanning electron microscopy, dynamic light scattering, differential scanning calorimetry, X-ray diffraction, and Fourier transform infrared analysis (FESEM, DLS, DSC, XRD, and FTIR, respectively), and their dissolution rates were then compared.

**Table 1:** Literature review on impregnation in different contexts (RESOLV).

Compound	Cosolvent	Material	Pressure (MPa)	Temperature	Time (h)	References
BSA-Conjugated Ag_2_S	Methanol	PVP, Silver nitrate, Sodium sulfide	27.6	160	0.5	^53^
Drug (Ibuprofen and Naproxen)	Methanol	PVP	20	40	0.25	^54^
Tetraphenylporphyrin (TBTPP)	Water	PVP	10.3–30.4	40–100	72	^55^
Poly(Heptadecafluoro decyl acrylate) PHDFDA	Ethanol	PMMA, PLA, NaCl	25	40	–	^44^
Ibuprofen	Methanol	PVP, PVA, SDS, BSA, PEG	20	40	0.5	^12^
Drug Amphotericin B	Methanol	DMSO, PVA	30.1	40	O.25	^56^
Poly[2-(3-thienyl)acetyl 3,3,4,4,5,5,6,6,7,7,8,8,8-tridecafluoro-1-octanate] (PSFTE)	–	SDS, NaCl	20.7	40	0.25	^48^
Chitin-HFIP	Ethanol	Naproxen, Tetraphenylporphyrin (TBTPP) Poly-L-lactic acid (PLA)	10.4–20.8	40–60	120	^57^
Poly(l-lactide) (PLLA)	–	SDS, Acetonitrile	34	50–60	–	^43^
Cortisone Acetate	Ethanol	PEG, Acetone	20	35	24	^58^
Fenofibrate (FNB)	Ethanol	SDS, HPMC	20	60	24	^45^
Paclitaxel	Ethanol	PVP	31	40	24	^46^
Catechin and Poly(l-lactide)	Ethanol	Tetrahydrofuran (THF),	26.5–32.5	60–100	8	^59^
Caffeic Acid Phenethyl Ester (CAPE)	Ethanol	DMSO, NaCl , PDA	17.3	30–37	24	^60^
Amiodarone hydrochloride (AMH)	–	PVP, HPMC	12.0–27	35–65	24	^7^
Gambogic Acid	–	DMSO	10.0–30	35–65	24	^47^
Sunitinib malate	–	PVA, HPMC	18.0–27	35–65	–	^18^

## Materials and methods

### Materials

PRH (CAS Number 19237-84-4) with 99% purity was purchased from Darupakhsh raw material production company (Temad) (Tehran, Iran). Aboughadareh Company (Shiraz-Iran) supplied CO_2_ with a 99.99% purity (Table 2).These compounds were used without further purification. Polyvinyl pyrrolidone with a molecular weight of 40,000 g/mol was purchased from Sigma Aldrich (Germany).

**Table 2:** The solute Prazosin hydrochloride structure and the corresponding physic-chemical profile.

Component	Formula	M_w_ (g mol^−1^)	CAS Number	Structure
Prazosin hydrochloride	C_19_H_22_ClN_5_O_4_	419.9	19237-84-4	
Carbon dioxide	CO_2_	44.01		
Polyvinyl pyrrolidone	(C_6_H_9_NO)_n_	40,000	9003-39-8	

### Methods

#### RESOLV apparatus and procedure

**Figure 1:**
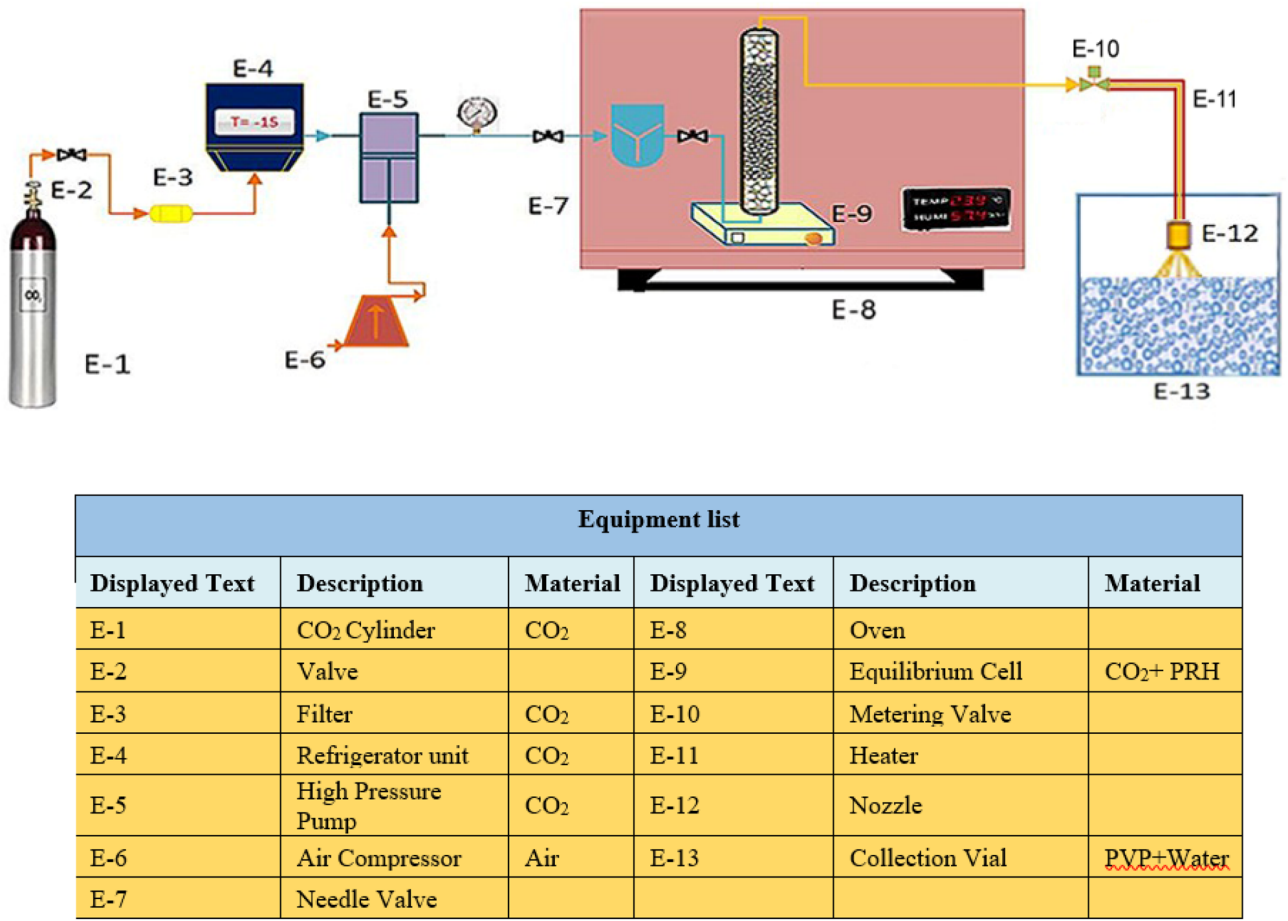
Experimental apparatus of the supercritical solution rapid expansion into a liquid solvent.

Figure 1 provides the schematic of the RESOLV method device, including units for extraction and precipitation. CO_2_ enters the extraction cell, which has an internal volume of 50 ml, to reach the desired maximum pressure (40 MPa). The components of RESOLV device include CO_2_ tank, Valve, filter, Air Compressor, Haskel pump (type—CA 91502, Burbank, CA, USA company), cooling and heating systems (refrigerator and oven, respectively), heather, orifice nozzle, participation sampler, flow meter, measuring instrument, and control panel.

First, 2 g of PRH is poured into the cell in a homogeneous form along with glass beads with a diameter of 2 mm to fill it. Then 1mL of ethanol as a cosolvent is added to the cell and the desired temperature (308, 318, and 328 K) and pressure (15, 20, 25 MPa) are adjusted. In the extraction device, two filters are placed at both ends of the cell, and the distance between the nozzle head and the solution is 2 cm. A refrigerator reduces the temperature of the CO_2_ to about 263 K at the process onset to prevent CO_2_ gas formation in the piston and its locking in the high-pressure pump. After that, CO_2_ enters the extraction cell with an internal volume of 50 ml using a high-pressure reciprocating pump to ensure the optimum pressure. A closely controlled oven with a ± 0.1 K temperature set the desired temperatures. After a static time (here set to 60 min), the saturated PRH-CO_2_ was transferred through a preheated 1/8" stainless steel tube and micrometer valve to an orifice nozzle to avoid clogging of the nozzle during the expansion phase. In addition, the dynamic time of rapid expansion was 180 min through the nozzle to solution with concentration 3 mg/ml (45 mg of PVP and 15 ml of deionized water). A few drops were then placed on the slide to form the layer. After that, it was placed in the oven to remove water from the composite and PRH samples were prepared for FTIR, DLS, FESEM, XRD and DSC analysis.

#### Particle characterization

The particles produced from the RESOLV process underwent characterizations by FTIR spectroscopic analysis, XRD, FESEM, DSC, and DLS. DSC analysis highlights the melting point values related to the original and processed drugs. Here, 5 mg of the sample is heated at a 10 °C/min heating rate over a 30–300 °C temperature range of temperature range in a standard aluminum pan under argon atmosphere with a 20 ml/min flow. The X-ray diffractometer of the original and processed patterns was checked utilizing CU-Ka radiation (λ = 0.154 nm under room temperature in 2 different values at a range of 5–80 θ, and this crystal structure, phase purity and average crystal size. FTIR analysis is used for the shape and position evaluation of absorption peaks. Thus, 3 mg PRH is coupled with spectral grade KBr (300 mg) by mortar and pestle, followed by the sample compression to obtain a KBr disk made on a Hitachi. FESEM analyses was used to investigate particle shape and surface profile. In FESEM imaging, gold–palladium alloy is employed to coat powder samples. For this purpose, in each run, a few drops of the suspended nanoparticle mixture were placed on a glass slide and placed in an oven to dry.

The nanoparticle size distribution was determined by DLS using a He.N_2_ laser with a wavelength of 623 nm and a power of 10 mW at a scattering angle of 90° as a light source. Approximately 1 mg of PRH was dissolved in 10 ml of deionized water prior to DLS analysis. Moreover, the loaded drug was calculated based on the method reported by Ameri et al.^38^ and reported in Table 3.

**Table 3:** Operation conditions of the RESOLV processes and quantitative results.

Run	P (MPa)	T (K)	Diameter of nozzle $\left( {\mu {\text{m}}} \right)$	Particle size (nm)	Predicted value (nm)	Drug loading (mg)
1	15	318	500	418.2	420.3	20.8
2	20	308	500	406.5	408.6	28.6
3	20	328	300	257.4	259.5	34.7
4	25	318	300	265.9	268.0	37.5
5	15	328	700	366.5	368.6	23.8
6	20	318	700	389.2	385.0	30.8
7	25	308	700	363.3	365.4	33.5
8	25	328	500	252.4	248.2	42.6
9	15	308	300	416.2	412.0	17.5

#### Experimental design of process parameters

Design of Experiments (DoE) method was used to determine the optimization values of parameters affecting PRH production. Parameter design contributes essentially to the Taguchi methodology to ensure higher quality with no increase in the costs. This research investigated DoE within RESOLV considering three orthogonal parameters in three levels (L-9). Preliminary tests were considered to choose the key factors and their relevant levels. The size of the nanosized particles was investigated by RESOLV according to the parameters of pressure in the range (15–25 MPa) and temperature (308–328 K) and nozzle diameter of (300, 500, 700 μm).

## Results and discussion

**Figure 2:**
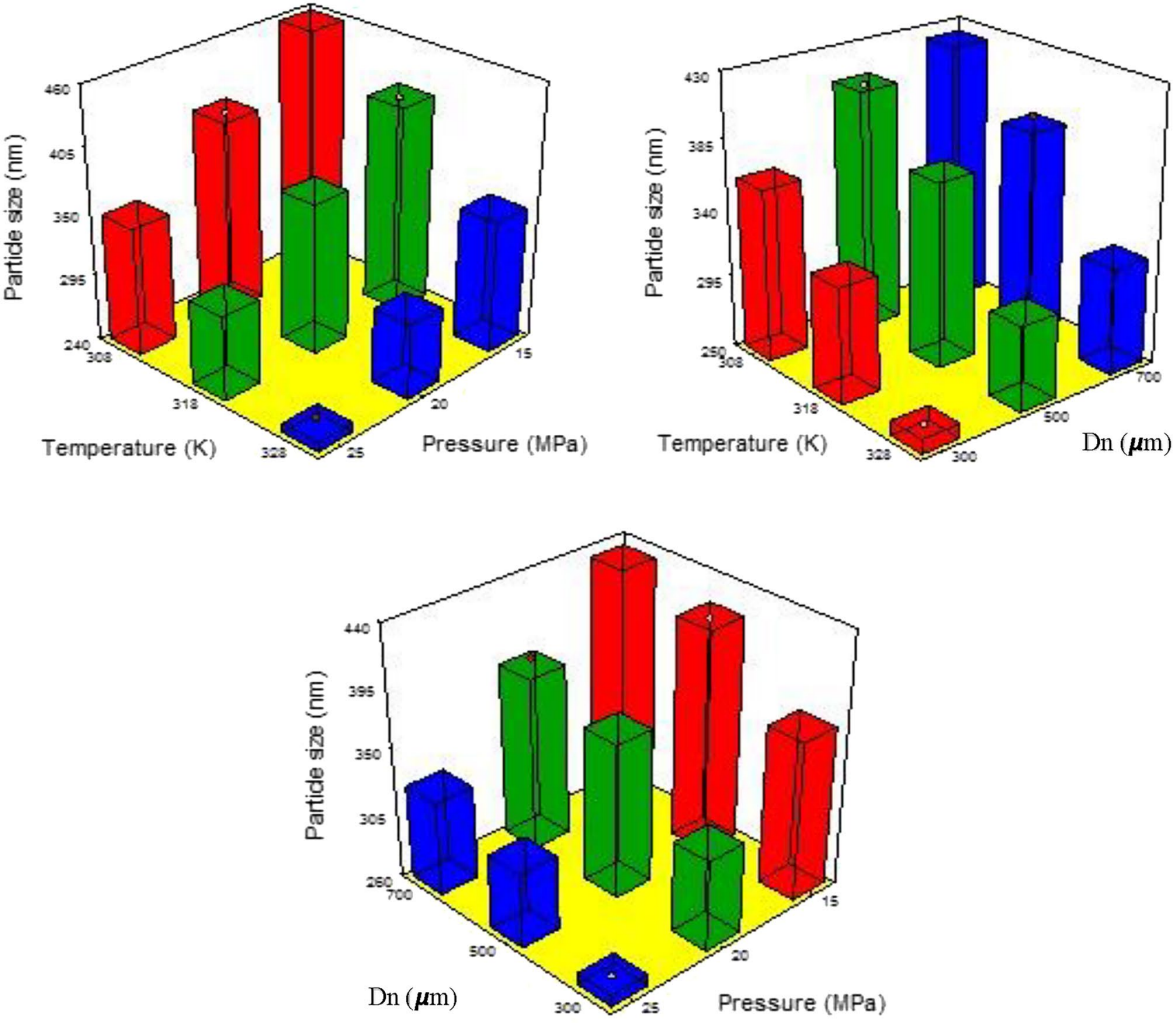
The particle size as a function of temperature–pressure and nozzle diameter (Dn).

**Figure 3:**
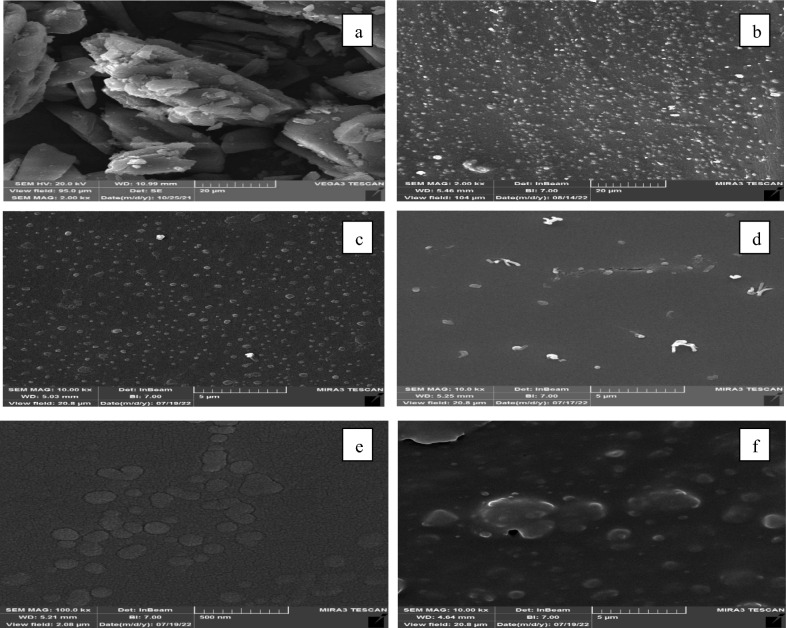
RESOLV FESEM images for various conditions: (**a**) original prazosin hydrochloride (PRH), (**b**) Run 1, (**c**) Run 2, (**d**) Run 3, (**e**) Run 4, (**f**) Run 7.

PRH solubility in SC-CO_2_ under 308–338 K temperature and 120–270 bar pressure ranges was investigated spectroscopically^61^.The obtained results showed the mole fraction between $1.59\times {10}^{-5}$ and $7.2\times {10}^{-5}$. RESOLV can be a favorable method for the formation of PRH micro and nanoparticles. Several parameters may influence particle size, particle size uniformity, morphology, and the medicinal compound's dissolution rate when using RESOLV. Temperature, pressure, nozzle diameter, polymer type, nozzle length, solvent content, and flow rate are the main influential parameters. This paper examined the effects of temperature (308–328 K) and pressure (15–25 MPa), nozzle diameter (300, 500, 700 μm), and type of PVP polymer on particle size. The conventional single-factor approach may be very costly and time-consuming at times. From design Taguchi (orthogonal arrangement L-9) it has been used for a better effect of the mentioned variables on the production of nanoparticles. The obtained outputs are presented in Tables 3 and 4 and Figs. 2 and 3. All measurements were repeated three times to obtain a more reliable result (values are reported by particle size uniformity).

**Table 4:** Taguchi method adequacy and ANOVA analysis for RESOLV.

Source	Std. dev	R-square	Adjusted R-square	Predicted R-square	p-Value	PRESS
Model	6.3	0.9979	0.9919	0.9592	0.006	1607.45
Source	Sum of squares	df	Mean square	F-value	Prob > F	
Model	39,282.62	6	6547.1	164.95	0.006	Significant
P	17,023.29	2	8511.64	214.45	0.0046	Significant
T	16,380.49	2	8190.24	206.35	0.0048	Significant
Nozzle diameter	5878.85	2	2939.42	74.06	0.0133	Significant
Residual	79.38	2	39.69			
Cor. Total	39,362	8

### Effect of operating conditions on particle size and particle size distribution in RESOLV

The RESOLV method included ANOVA utilizing the experimental results in Table 3 to examine the importance of pressure (15, 20 and 25 MPa), temperature (308, 318, 328 K) and nozzle diameter (300, 500 and 700 μm). Hence, parameters with smaller p- (*p* < 0.05) and larger F-values had a significant impact on the RESOLV process. Table 4 highlights the statistical significance of pressure (*p* = 0.0046), temperature (*p* = 0.0048), and nozzle diameter (*p* = 0.0133) in terms of reduced particle size. Higher effectiveness of pressure and temperature than nozzle diameter in reducing size is also confirmed by the results. Various statistical indices, including coefficient of determination (R^2^), adjusted and predicted coefficients of determination (R^2^_adj_ and R^2^_pred_, respectively), sufficient accuracy, and coefficient of variation (CV), were utilized for statistical significance investigations. Values of R^2^ (0.9979), R^2^_adj_ (0.9919), and R^2^_pred_ (0.9592) can highlight the importance of the method and predict the model's adequacy. Pressure, temperature, and nozzle diameter are the most effective parameters explaining 0.9919 of the total size reduction variation, as shown by the adjusted model's R^2^ value. The resulting value of sufficient accuracy (34.12, i.e., more than 4) shows an excellent signal-to-noise ratio for movement in the designed space. The low CV value (4.06) confirms the test reliability. ANOVA was utilized to analyze the experimental results obtained for the pressure, temperature, and nozzle diameter impacts on PRH particle size (Table 4) to determine the best particle size-parameters connection.

CV shows the exact degree of comparison of treatments, indicating that the experimental results are appropriately accurate and reliable by CV = 3.9. The estimated R^2^ for PRH particle size is 0.9979. As shown in Table 4, the statistical model can justify 99.79% of the response changes. The predicted and adjusted R^2^ show reasonable agreement, with respective values of 0.9592 and 0.9919. The large value of determined coefficients (R^2^_adj_ = 0.9919) indicates a very significant model break. The predicted residual error sum of squares (PRESS = 1607.45) presents a measure of model fit for the search points in the design and can be estimated by squaring the difference between the actual predicted values at each point and the sum of squares across the total set of points. Lower PRESS values represent a better model fit into the data points.

### The effect of parametric conditions

All the facts presented above support Taguchi as an appropriate model for the present research. Important conditions and parameters are pressure, temperature and nozzle diameter. The design responses for the independent parameters, which include the associations of particle size and parameters, are shown in Fig. 2. Data in Table 3 attribute the highest importance to pressure in reducing the size of particles. When the pressure increases from 15 to 25 MPa, the particle size decreases from 418.2 to 252.4 nm, which is clearly evident in Fig. 2.The detailed data and discussions on the pressure impact on the size of particles were previously provided. Noteworthy, with increasing pressure, the strength of the solvent increases, consequently enhancing the nucleation rate and the production of fine particles. The temperature impact on the RESOLV process was studied as a thermodynamic factor. The graphs in Fig. 2, show the temperature, pressure, and nozzle diameter impacts on the size of particles. The first two parameters show an interactive effect on particle size, making the assessment of temperature effects on the process more difficult than pressure. An increase in the temperature leads to a reduction in the SC-CO_2_ density, consequently decreasing the solvent power. On the contrary, an increased sublimation vapor pressure can be observed at higher temperatures, increasing the supercritical fluid's solubility. Therefore, increasing temperature alters SC-CO_2_ solubility according to the impacts imposed by solvent density and sublimation vapor pressure^18,62^. As highlighted by the obtained data, increased temperature to 318 K results in larger particles, probably due to the double effects on the particle size. At this temperature, the solvent power effect overcomes the sublimation vapor pressure. Overall, temperature's positive effects can be considered as a higher sublimation vapor pressure under greater temperatures. Another parameter that can affect the particle size production of processed PRH is the nozzle diameter. Experimental tests were carried out with different nozzle diameters: 300, 500 and 700 microns. As shown in Fig. 2, the smallest particles were obtained with a 300 μm diameter nozzle. At a temperature of 308 K with a smaller nozzle diameter (Dn = 300 μm), increasing the nucleation rate increases the risk of coagulation of smaller particles and eventually larger particles are formed. However, with the PVP solution, coagulation did not occur. By increasing the nozzle diameter to 700 µm, a slight increase in particle size was observed. Table 3 shows that at a temperature of 328 and 318 K, the smaller the nozzle diameter used in the RESOLV method, the smaller the size of the particles produced. The optimum values of temperature 308 K, pressure 25 MPa and nozzle diameter 300 μm were determined. These values were predicted for 202.5 nm particles. Therefore, the average particle size was found to be 217.4 nm, which was close to the predicted value.

### PRH characterization

**Figure 4:**
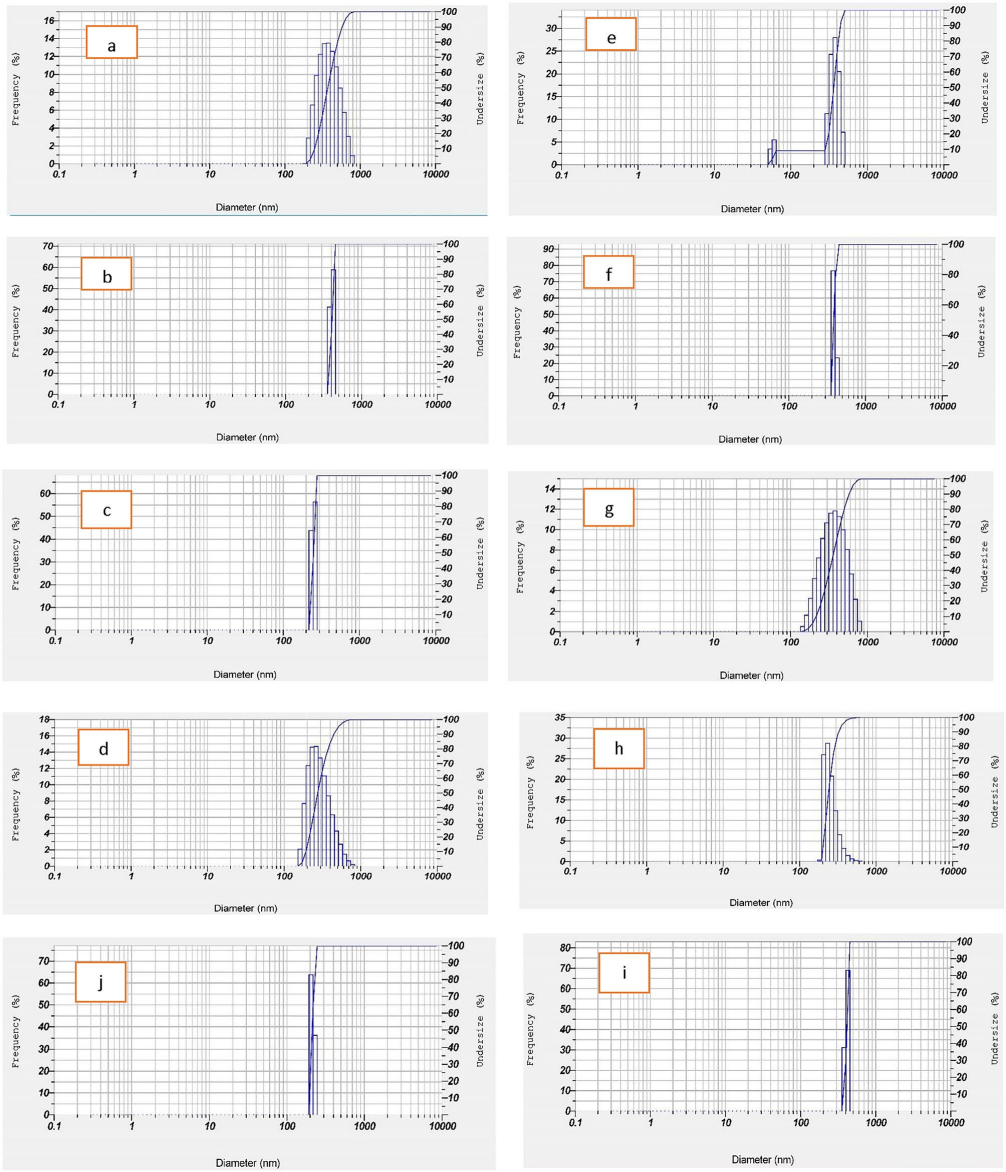
DLS graphs for (**a**) Run1, (**b**) Run 2, (**c**) Run 3, (**d**) Run 4, (**e**) Run5, (**f**) Run6, (**g**) Run7, (**h**) Run 8, (**i**) Run9, (**j**) Optimum condition.

**Figure 5:**
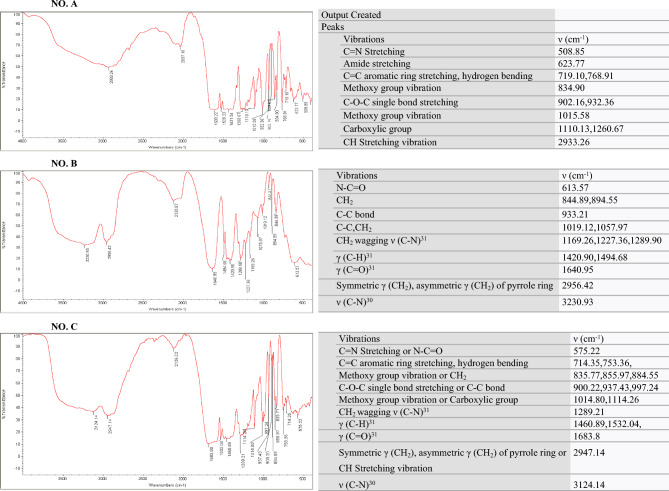
Comparison of the FTIR spectrum of (A) the original PRH (top), (B) PVP, (C) RESOLV processed PRH-PVP, (Optimum conditions).

The structures and morphologies of the original PRH and the RESOLV-PVP nanoparticles were determined by FESEM, and the results are shown in Fig. 3. According to the SEM results of the original PRH, the original PRH consists of irregularly shaped crystals (Fig. 4). The FESEM results show the particle size reduction in the processed samples well with the RESOLV method in Figures b and c. PRH and polymer-drug molecules were mixed in the matrix and precipitated together, and Figures d, e and f show that PRH is trapped in the polymer. (Fig. 3). The analysis of the FTIR spectra recorded in Fig. 5 is related to PRH. By studying the FTIR diagram of the obtained sample and comparing it with the diagrams of two substances, drug and PVP, it is possible to identify the peaks in the total sample of the peaks and those in the two samples of PVP and drug, which are displayed with similar colors in the table, confirming the presence of these materials in the sample.

**Figure 6:**
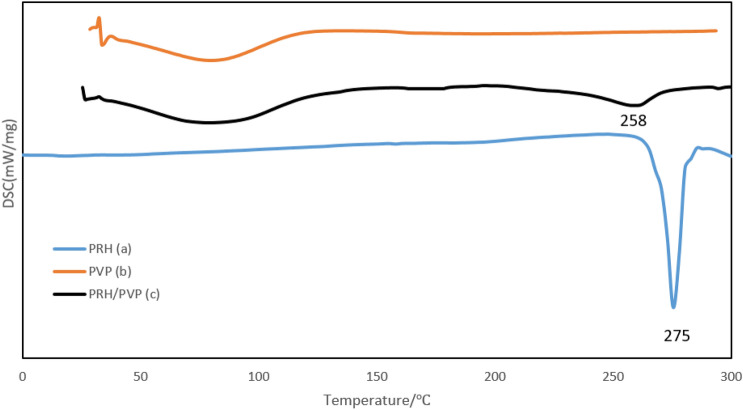
Pre- and post-RESOLV process DSC analysis results: (**a**) the original PRH; and (**b**) original PVP (C) RESOLV-SC processed PRH, (Optimum conditions).

Figure 6 shows the DSC analysis results for pure PRH, pure polymer, and the obtained sample. Pure PRH shows a 275°C melting point temperature (T_m_), which is exothermic according to the corresponding peak characteristic, indicating the parent PRH's crystalline nature and highlighting good agreement with previous findings^5,63^.

DSC analysis of pure PVP in Fig. 6 shows that the exothermic transition occurs in the range of 55 to 80 °C, which corresponds to the diffusion of moisture at the adsorption temperature. According to the DSC results of the obtained sample in Fig. 6, there is a slight change in the position and intensity of PRH particles' exothermic melting peak to 258.3° Celsius utilizing PVP, possibly associated with the particles' smaller size and amorphous configuration (Pathak et al.^12,46^). There is also a difference melting point and peak intensity for RESOLV in PVP solution, which confirms the system's reduced size and amorphous nature.

The DLS graphs of PRH nanoparticles under different conditions (runs 1, 3, 4, 5, 6 and 7) are shown in Fig. 4. The DLS and FESEM results are in good agreement. Based on Fig. 3, the thinnest particle size distribution is related to the run at the lowest temperature. The DLS and FESEM results showed that the micro-scale particle size of unprocessed PRH changed to the nano-scale particles after the RESOLV process. The average particle size of the processed PRH (runs 1, 3, 4, 5, 6 and 7) was determined by DLS analysis to be 372.8, 246.98, 562.2, 328.8, 259.2 and 378.7 nm.

**Figure 7:**
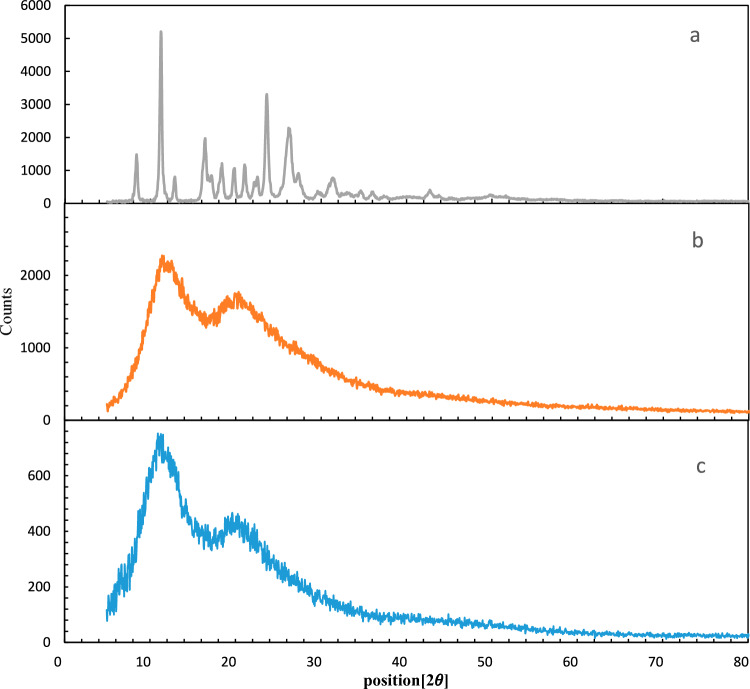
XRD for (**a**) the original PRH; (**b**) original PVP; (**c**) RESOLV-SC processed PRH, (Optimum conditions).

XRD analysis was used (Fig. 7) to evaluate the crystal structure of PRH, indicating the original PRH to be well crystalline with clear peaks at 5–80 of 2θ diffraction angles. As presented in Fig. 7, RESOLV XRD analysis shows that the processed drug has less crystallinity than the original particles, potentially because of their particle size reduction. However, no obvious peak indicating the crystalline nature of the processed samples was seen, while there was a broad peak indicating the samples' amorphous nature. These results prove the effectiveness of RESOLV in placing PRH in amorphous state.

### Optimum conditions

The optimum values of the process parameters were determined to obtain the smallest particle size using the Taguchi method implemented in the Design Expert software. These values were determined to be a temperature of 308 K, a pressure of 25 MPa, and a nozzle diameter of 300 μm. These values were predicted to yield particles of 202.5 nm in size. The Taguchi method was used to evaluate the accuracy and validity of the optimization method through experiments, and the average particle size was found to be 217.4 nm, which was very close to the predicted value.

## Conclusion

This study used the RESOLV method for the first time to control the size and also the size distribution of PRH as a water-insoluble drug. The effects of temperature (308–328 K), pressure (15–25 MPa) and nozzle diameter (300, 500, 700 μm) on particle size were investigated. PRH drug nanoparticles were identified using FTIR, XRD, DSC, FESEM analyses. FTIR and XRD analyzes showed that no changes occurred in the chemical structure of PRH. FESEM results show the reduction of particle size by RESOLV method. According to the DSC results of the sample obtained, there is a slight change in the position and intensity of the exothermic melting peak of PRH particles up to 258.3 °C using PVP, which is probably related to the smaller particle size and amorphous configuration. Besides, the pressure, temperature, nozzle diameter impacts on the morphologic profile, particle size distribution were investigated and underwent optimizations by the design method. In the Taguchi method, the lowest and highest values achieved are 248.2 and 420.3 nm. The lowest value calculated by RESOLV is 252.4 nm and the highest value is 418.2 nm. This experiment is in good agreement with Taguchi's design. A particle size of 202.5 nm was predicted by the Taguchi method. The average particle size was 217.4 nm, which was very close to the expected value when the Taguchi method was used to evaluate the accuracy and validity of the optimized point.

## Data Availability

The datasets used and/or analyzed during the current study are available upon reasonable request from the corresponding author.
